# Metal organic vapour-phase epitaxy growth of GaN wires on Si (111) for light-emitting diode applications

**DOI:** 10.1186/1556-276X-8-61

**Published:** 2013-02-07

**Authors:** Damien Salomon, Amelie Dussaigne, Matthieu Lafossas, Christophe Durand, Catherine Bougerol, Pierre Ferret, Joel Eymery

**Affiliations:** 1 Equipe mixte CEA-CNRS-UJF “Nanophysique et semiconducteurs”, SP2M, UMR-E CEA/UJF-Grenoble 1, INAC, Grenoble, 38054, France; 2 CEA-Leti, MINATEC campus, Grenoble, 38054, France; 3 Equipe mixte CEA-CNRS “Nanophysique et semiconducteurs”, Institut Néel-CNRS, 25 rue des Martyrs, Grenoble, Cedex 9 38042, France

**Keywords:** Nitrides, Nanowires, LED, MOVPE, 81.07.Gf, 81.05.Er, 85.60.Jb

## Abstract

GaN wires are grown on a Si (111) substrate by metal organic vapour-phase epitaxy on a thin deposited AlN blanket and through a thin SiN_*x*_ layer formed spontaneously at the AlN/Si interface. N-doped wires are used as templates for the growth of core-shell InGaN/GaN multiple quantum wells coated by a p-doped shell. Standing single-wire heterostructures are connected using a metallic tip and a Si substrate backside contact, and the electroluminescence at room temperature and forward bias is demonstrated at 420 nm. This result points out the feasibility of lower cost nitride-based wires for light-emitting diode applications.

## Background

III-Nitride semiconductor nanowires (NWs) have recently attracted great interest due to their potential applications including light-emitting diodes (LEDs), lasers, photodetectors, gas sensors and solar cells [1-5]. The direct growth of NWs on conductive substrates benefits from a direct electrical backside contact that can considerably simplify the device processing. In this context, silicon wafers present several attractive advantages to be employed as n- or p-type conductive substrates such as scalability (up to 12 in.), good thermal conductivity and low cost. The planar growth of GaN on Si substrates is challenging because of the large lattice and thermal dilatation mismatches that create high dislocation densities and residual strains. The NW geometry is known to improve these two drawbacks by decreasing the dislocation density along the wire length and releasing the strain with the free surface relaxation. The growth of GaN NWs on Si (111) has been mainly developed by catalyst-free molecular beam epitaxy (MBE) using an intermediate interfacial AlN layer to improve the epitaxial relationships [6,7]. Such nanowires exhibit excellent optical properties and have been successfully integrated in LED devices [8]. Metal organic vapour-phase epitaxy (MOVPE), which is widespread in the industry for planar growths, has been used to address the growth of catalyst-free GaN wires [9-11]. But surprisingly, the MOVPE growth of GaN wires on Si (111) substrate has been reported only recently using deposited Al [12] and AlN [13] intermediate layers. The roles of these thin layers on the epitaxial relationships between the substrate and the wires and their impact on the LED electrical injection have not been reported yet.

These two points will be studied in this paper by growing n-doped GaN wires by MOVPE on a thin AlN layer deposited on n-type Si (111) substrates. The epitaxial relationship of the wire with the AlN/Si interface will be confirmed by X-ray diffraction (XRD) and high-resolution transmission electron microscopy (HRTEM). Then, we will demonstrate that such wires can be used as a template to build a complete LED heterostructure based on InGaN/GaN quantum wells grown on the side facets. The electrical properties of single bright-violet electroluminescent wires will be studied to demonstrate the interest of the direct injection from the Si substrate.

## Methods

The growth is performed in a close-coupled showerhead MOVPE reactor. Si (111) substrates are deoxidized before growth in a 10% HF solution for 1 min. The substrate surface is then cleaned and smoothed with a 20-min bake at 1,100°C and 100 mbar under H_2_. The direct MOVPE deposition of GaN on Si at high temperature using trimethylgallium (TMGa) results in the formation of hollows in the substrate due to strong chemical reactions [14]. Therefore, unlike to the growth on sapphire, the Si substrate has to be protected first by a thin AlN buffer layer deposited at high temperature using trimethylaluminium (TMAl) and NH_3_ precursors. Under such growth conditions, the polarity of the AlN layer is Al-polar [15], and its thickness has no significant influence on the later GaN wire growth. According to our previous work [11], a thin SiN_*x*_ layer is first deposited on the AlN surface to prevent GaN planar growth. Self-assembled catalyst-free GaN wires are then grown for 500 s using TMGa and NH_3_ precursors with a low V/III ratio (approximately 20) and silane injection to favour the vertical growth [16].

## Results and discussion

**Figure 1:**
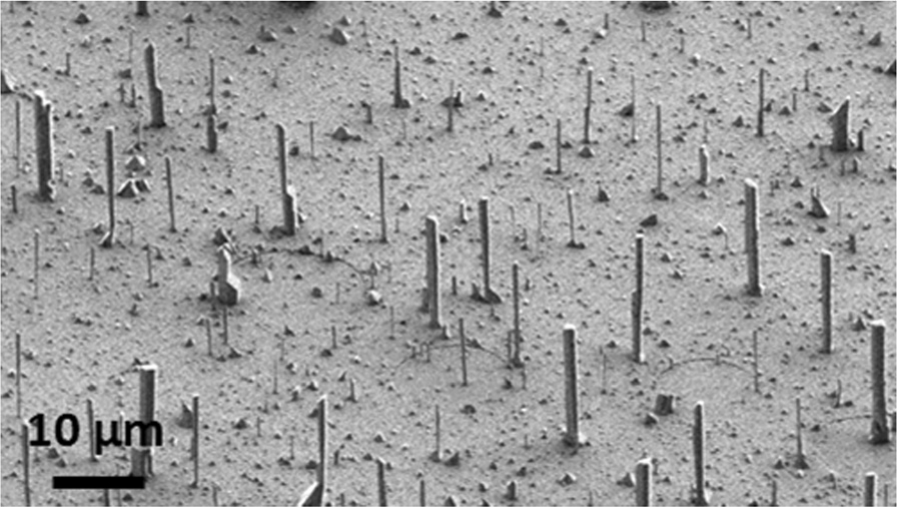
**SEM picture of GaN wires.** 45° tilted view of GaN wires grown by MOVPE on Si (111) with an intermediate AlN layer.

Figure 1 shows a typical 45° tilted SEM image of the resulting vertically aligned GaN wires. They exhibit an irregular hexagonal cross section and a quite large dispersion in length and diameter. Due to the very low wire density (approximately 10^6^ wires/cm^2^), specular X-ray reflectivity (not shown in this paper) allows measurement of the total layer thickness on top of silicon. Well-contrasted interference fringes corresponding to a thickness of 25 ± 0.5 nm are measured close to the target value for the AlN layer. HRTEM cross sections have shown no significant planar growth on the surface. This is in agreement with the deposition of the SiN_*x*_ passivation layer on top of AlN, as already observed for the growth of GaN wires on sapphire [11].

**Figure 2:**
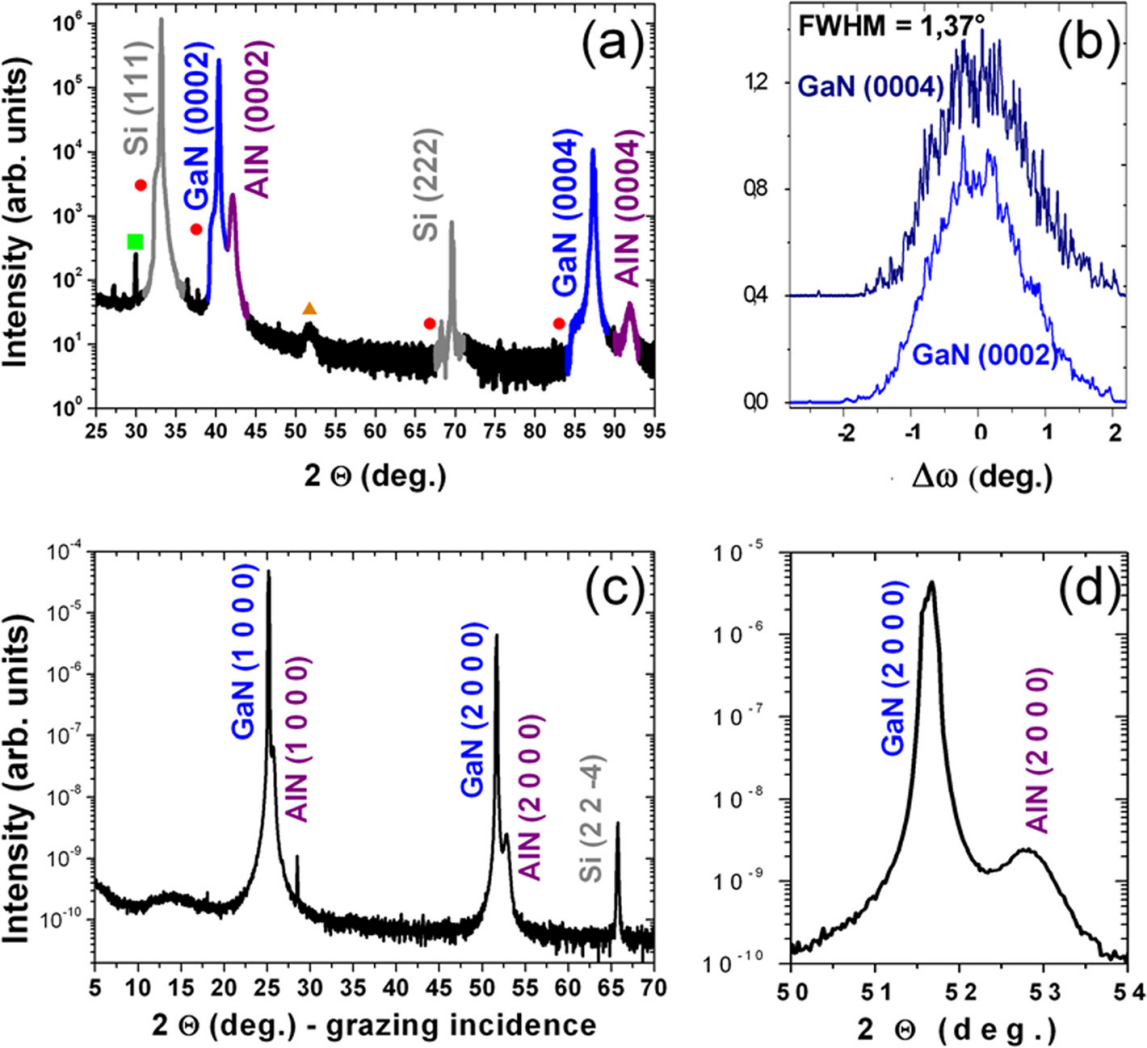
**X-ray diffraction measurements of GaN wires grown on Si (111) with an intermediate AlN layer.** (**a**) Symmetric Θ-2Θ scan performed on a laboratory setup (approximately 0.179 nm Co-wavelength) and indexed with Si, GaN and AlN Bragg K_α1_ reflections. Dots and squares correspond respectively to the K_α2_ and K_β_ excitation wavelengths. The broad and low intensity peak around 51° (see the triangle) is attributed to a diffraction tail of the Si substrate. (**b**) Rocking curves (Δ*ω*-scan) of the GaN (0002) and (0004) peaks. (**c**,**d**) Grazing incidence X-ray diffraction performed at ESRF along the $\left[22\overset{￣}{4}\right]$ silicon direction (approximately 0.1203 nm wavelength and 0.18° incidence).

**Figure 3:**
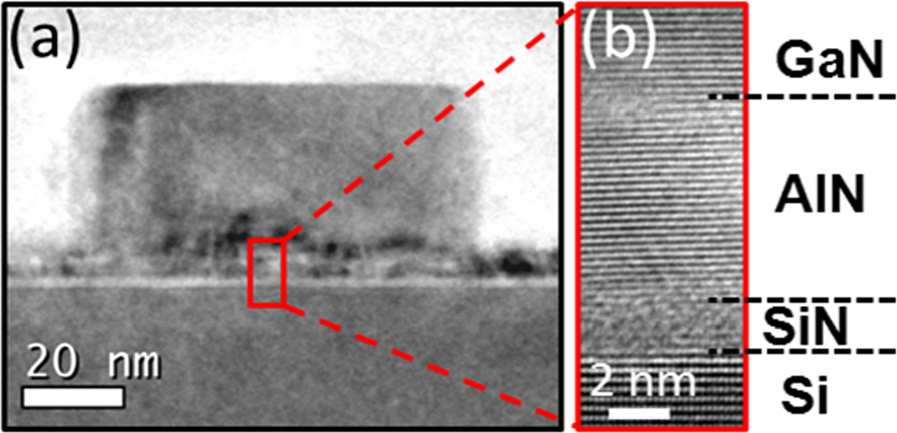
**HRTEM imaging of the GaN/AlN/Si interface (a,b).** Observation along the $\left[10\overset{￣}{1}0\right]$ zone axis showing the materials stacking.

The structural properties of the wires were first investigated by laboratory XRD using symmetric (Θ-2Θ) and rocking (*ω*) scans. Figure 2a shows the Θ-2Θ diffraction pattern of the as-grown samples with a cobalt radiation source. The GaN (0001), AlN (0001) and Si (111) Bragg peaks are indexed, indicating a GaN wire growth orientation along the *c*-axis. The disorientation of the GaN wires was investigated by the Δ*ω* rocking curves of the GaN (0002) and GaN (0004) Bragg peaks. As shown in Figure 2b, the 1.37° full width at half maximum of the peaks gives an estimation of the tilt disorientation. This relatively large value compared to the previous measurement on sapphire (0.61°) [11] can be attributed to the AlN buffer layer epitaxial quality and to the nucleation on the defects. HRTEM cross-section observations have been performed to investigate the epitaxial relationship in between the GaN wire/AlN buffer/Si substrate. The observation was made with a JEOL 3010 (JEOL Ltd., Tokyo, Japan) operating at 200 kV along the $\left[10\overset{￣}{1}0\right]$ zone axis. Figure 3a shows the base of a GaN wire grown on Si with an AlN buffer layer of 10-nm nominal thickness. As shown by the detailed view of Figure 3b, four distinct layers are observed. A 2-nm-thick amorphous (or nanocrystallized) layer is observed directly on top of the Si substrate. This layer can be attributed to the spontaneous SiN_*x*_ formation resulting from the high-temperature growth of the AlN buffer on silicon as already reported by Radtke et al. [15]. The AlN seeds probably nucleate through this non-continuous thin silicon nitride layer, and a planar growth develops laterally to form an almost single-crystalline AlN epitaxial layer for further growth. To confirm these assumptions, the in-plane epitaxial relationships have been studied at the European Synchrotron Radiation Facility (ESRF, Grenoble, France) on the French BM32 CRG beamline with a 0.1204-nm wavelength. Grazing incidence X-ray diffraction (GIXRD) has been performed with 0.18° incidence to check the AlN epitaxy on SiN_*x*_/Si (111). The usual orientations [17] have been measured corresponding to the AlN $\left[11\overset{￣}{2}0\right]$ //Si $\left[2\overset{￣}{2}0\right]$ and AlN $\left[10\overset{￣}{1}0\right]$ //Si $\left[22\overset{￣}{4}\right]$ alignments. These measurements confirm also the complete registry of GaN wires with the AlN layer (see for example the scans along the Si $\left[22\overset{￣}{4}\right]$ direction shown in Figure 2c,d). The AlN layer has been formed at high temperature (approximately 1,100°C) in the 10- to 50-nm range to sufficiently protect the surface and maintain the epitaxy. The study of the epitaxial relationship at lower growth temperature and different thicknesses could be interesting in further studies.

These n-doped GaN wires (in the 10^18^-cm^−3^ range estimated from the width of photoluminescence peaks [11]) can be used as templates for the growth of a complete LED structure combining unintentionally doped InGaN/GaN multiple quantum wells (MQWs) on the side facets and convenient doping junctions. Nominal In_0.18_Ga_0.82_N (1 nm)/GaN (10 nm) MQWs are grown using trimethylindium (TMIn), triethylgallium (TEGa) and NH_3_ as described in [18] and coated by a p-GaN layer doped in the 10^17^-cm^−3^ range using TMGa, NH_3_ and bis(cyclopentadienyl)magnesium (Cp_2_Mg).

**Figure 4:**
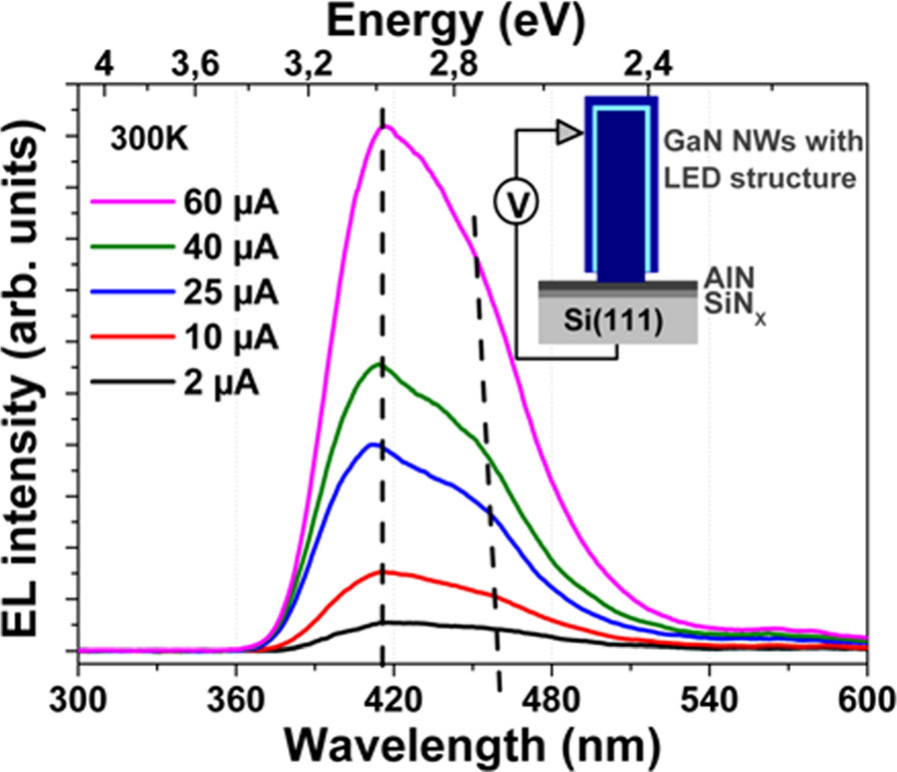
**Electroluminescence measurements.** Electroluminescence spectra of a single InGaN/GaN core-shell wire LED structure measured at 300 K with a metallic tip (> 20 V) for 2, 10, 25, 40 and 60 μA. The inset shows a schematic view of the contact.

Electroluminescence (EL) measurements shown in Figure 4 were carried out on a probe station under continuous-wave (CW) operation and ambient conditions on single standing LED wires. As shown in the inset, the current is injected into the wires from a 2-μm radius metallic tip on the external sidewall p-doped layer and collected through the n-core wire, the AlN/SiN_*x*_ interface and the 275-μm-thick Si substrate (phosphorus-doped with a 10^−2^ Ω cm resistivity). EL spectra for different CW currents ranging from 2 to 60 μA have been obtained for high voltage bias between 40 and 20 V. This high turn-on voltage (*V*_on_) can be attributed to the electrical injection that involves two barriers coming from the wire/Si and wire/tip interfaces in addition to the resistive behaviour of the Si substrate. The AlN layer has a bandgap of approximately 6.2 eV and a conduction band offset with respect to Si (GaN) estimated to be approximately 2.3 (2.1) eV [19,20]. These barriers do not explain however the very high *V*_on_ of the device. For a comparison, the electron injection through a thick AlGaN/AlN epilayer has been reported to be only about 4 V [21]. Therefore, the high turn-on voltage can be mainly attributed to the contact between the metallic tip and the p-doped part of the structure. This assumption has been confirmed by the connection of an assembly of wires by indium titanium oxide exhibiting *V*_on_ ~ 10 V [13]. The EL spectra exhibit a violet emission centred at 420 nm and no defect band (the usual yellow band being close to 550 nm). These results demonstrate the possibility to make a wire-based LED device on silicon by MOVPE. A weaker low-energy contribution is also measured at 460 nm. The origin of these two contributions has been assigned by cathodoluminescence mapping [5] to the presence of both radial (420 nm) and axial (460 nm) MQWs inside the wires (note that these luminescence peaks are also measured for wires that are not coated by the Mg-doped GaN shell). The 40-nm shift of the wavelength could be attributed to the variations of the In composition, well thickness and/or to the influence of the electric field [18] corresponding to the *c*- or *m*-plane MQW growth orientations. The influence of the internal electric field on the luminescence wavelength is negligible due to the small thickness of the wells (estimated to be 1 nm by TEM observations). This point is also confirmed by the lack of any significant peak shifts with increasing current density. The variation of In composition and well thickness is therefore the main origin of this wavelength shift in a single wire. Moreover, for different wire diameters, no significant change in the emission wavelength has been measured. It can be explained by the quite large thickness and low density of the wires compared to MBE samples where the In incorporation in the MQWs has been shown to vary strongly for a small diameter (140 to 270 nm for the 400-nm period) [22].

## Conclusions

In summary, we have shown the possibility to grow self-assembled vertically aligned GaN wires on the Si (111) substrate using a thin AlN intermediate layer. The epitaxial relationship of the GaN wires/AlN/Si (111) has been studied by XRD and GIXRD. As shown by HRTEM observations and in agreement with literature, the high growth temperature of AlN leads to the formation of an amorphous (or nanocrystallized) SiN_*x*_ layer between the Si and the AlN that does not affect the epitaxial relationship. The wires were then used as templates for the growth of a complete LED structure, and the electrical continuity between the Si substrate and the n-GaN wire core allows the injection of electrons in the structure using a backside contact. A violet electroluminescence at 420 nm of single wires has been demonstrated and provides a low cost wire-based LED alternative for optoelectronic devices on Si when the voltage threshold will be reduced.

## Competing interests

The authors declare that they have no competing interests.

## Authors' contributions

DS carried out the sample growths, SEM imaging and XRD measurements and drafted the manuscript. AD and ML participated in the sample growth. CB carried out the TEM imaging. JE performed the grazing incidence XRD. CD, PF and JE participated in the supervision of the Ph.D. thesis of DS. All authors drafted, read and approved the final manuscript.
